# Pedestrian Mortality in Espírito Santo: A Time Trend Analysis from 2009 to 2020

**DOI:** 10.3390/epidemiologia7040091

**Published:** 2026-07-01

**Authors:** Rayssa Ribeiro da Silva, Fernando Rocha Oliveira, Déborah Ferreira de Carvalho Rodrigues, Lucas de Souza Soares, Raiza Brito Cipriano, Yasmin Neves Soares, Paulo André Stein Messetti, Italla Maria Pinheiro Bezzera

**Affiliations:** 1 Public Policy Laboratory, Higher School of Sciences of the Santa Casa de Misericórdia de Vitória (EMESCAM), Vitória 29045-402, ES, Brazil; rayssa.silva@edu.emescam.br (R.R.d.S.); deborah.rodrigues@edu.emescam.br (D.F.d.C.R.); lucaszsar@outlook.com (L.d.S.S.); ciprianoacademic@gmail.com (R.B.C.); yasminnevessoares@hotmail.com (Y.N.S.); 2 Program in Public Policies and Local Development—EMESCAM, Vitória 29045-402, ES, Brazil; paulo.messetti@emescam.br (P.A.S.M.); italla.bezerra@emescam.br (I.M.P.B.)

**Keywords:** traffic accidents, mortality, epidemiology

## Abstract

Background/Objectives: Traffic accidents are a significant public health issue. Pedestrians are considered the most vulnerable victims, showing the highest mortality rates. Thus, mortality rate indicators reflect the effectiveness of policies and safety measures applied to urban mobility. Therefore, the study objective is to identify trends in pedestrian mortality to foster an understanding of the local reality and to propose effective interventions for the safety and mobility of this population. Methods: This ecological time-series study used secondary data from the Unified Health System Information System (DATASUS) on all traffic accident-related deaths in Espírito Santo, Brazil, from 2009 to 2020. Data were classified according to the 10th Revision of the International Classification of Diseases (ICD-10). Variables included sex (male; female), age group (in years: 0 to 80+), and victim type (pedestrian). Mortality rates were logarithmically transformed (base 10), and the Prais–Winsten regression model was employed using STATA 13.0. Results: A total of 1969 traffic accident-related deaths were recorded. Males accounted for 75% of deaths, individuals of mixed race (pardo) represented 57%, and 72% were unmarried. A significant reduction in mortality rates was observed across age groups, especially among individuals aged 0–24 years. Mortality trends remained stationary only among individuals aged 80 years and older. Overall, the mortality rate decreased throughout the study period, from 5.51 to 1.69 deaths per 100,000 inhabitants. Conclusion: Pedestrian mortality rates from traffic accidents in Espírito Santo showed a decreasing trend, particularly among children and young people, while remaining stable among the elderly.

## 1. Introduction

Pedestrian accidents represent one of the most critical road safety issues worldwide, characterized by collisions between vehicles and individuals traveling on foot. As the second most vulnerable group in traffic, behind only motorcyclists, pedestrians exhibit significantly higher mortality rates in traffic crashes, accounting for 21.7%, compared to 12.3% among vehicle occupants. This vulnerability is exacerbated in middle-income countries, where rapid and unplanned urban growth and increased motorization coexist with deficient infrastructure and weak enforcement [1,2,3].

In Brazil, despite legislative advances such as the enactment of the Brazilian Traffic Code (1998) and the “Dry Law” (DL-2008), pedestrian mortality remains high. Accelerated urbanization, combined with policies that promote motorization through easy access to credit, has heightened the risk of fatal collisions, especially in metropolitan areas. Although regional studies point to positive effects of these measures, outcomes remain unequal across states and municipalities, reflecting historical disparities in urban infrastructure distribution and local governance capacity [4,5].

These inequalities are also evident in the economic burden of traffic accidents on the healthcare system. Between 2012 and 2022, traffic accidents in Brazil generated a cumulative cost exceeding USD 382 million for the Unified Health System (Sistema Único de Saúde [SUS]), with expenditures particularly concentrated in the Southeast region. Pedestrians, in addition to being among the most severely affected in terms of lethality and injury severity, also require longer hospital stays and rehabilitation periods, thereby increasing healthcare costs [6].

This economic burden is further compounded by factors such as poorly maintained sidewalks, lack of adequate signage, and insufficient traffic light timing, which are conditions that disproportionately affect older adults and people with disabilities [6].

At the state level, Espírito Santo exemplifies many of the structural and behavioral challenges previously discussed. In 2022, a total of 33,508 traffic accident victims were recorded in the state, of which 2.4% resulted in death and 97.6% sustained non-fatal injuries [7,8]. These largely preventable outcomes reflect persistent structural deficiencies related to road and vehicle quality, as well as insufficient enforcement, reckless behavior by both drivers and pedestrians, and gaps in traffic education and user behavior [8,9].

Given that pedestrians are the most vulnerable group in traffic accidents, this study aims to analyze the temporal trends of traffic accidents involving pedestrians in the state of Espírito Santo between 2009 and 2020.

## 2. Materials and Methods

### 2.1. Study Design and Study Population

This is an ecological study that analyzes the epidemiological profile of traffic accident-related pedestrian deaths in the state of Espírito Santo, Brazil, between 2009 and 2020.

**Figure 1:**
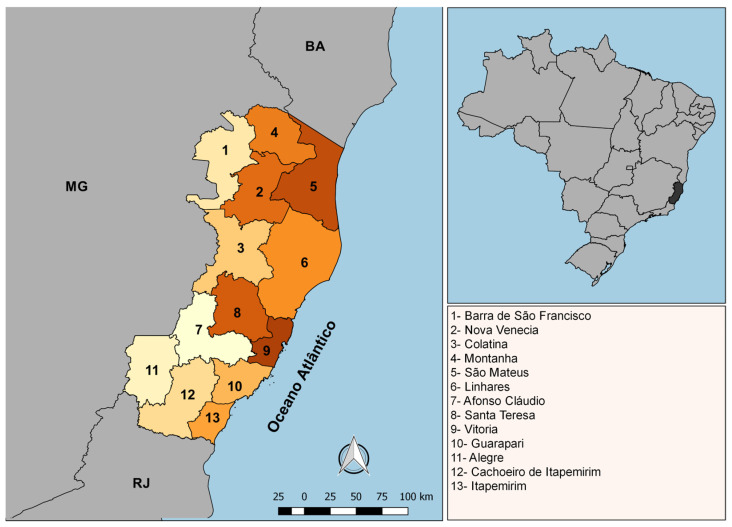
Map of Brazil with the representation of the state of Espírito Santo according to the IBGE microregions’.

Espírito Santo is located in the southeastern coastal region of Brazil (Figure 1), with a population of 3,833,712 inhabitants, a population density of 83 inhabitants per square kilometer, 78 municipalities, and a total of 2,248,960 registered vehicles [10].

### 2.2. Study Variables

The study data were obtained from the Sistema de Mortality Information System (SIM), made available by the Department of Informatics of the Unified Health System (DATASUS). This department provides information to support evidence-based analyses of the Brazilian health situation and to develop health action programs [11]. Data on deaths from all traffic accidents were selected, for which causes of death correspond to codes V01, V02, V03, V04, V05, V06, V08, and V09 from Chapter XX of the Tenth Revision of the International Classification of Diseases and Related Health Problems (ICD-10) [12]. Population information was collected from Brazilian Institute of Geography and Statistics (IBGE) for the studied years. The analyzed variables included sex (male, female), age group (in years: 0 to 80 and over), and victim type (pedestrian). The 2009–2020 period was chosen because it represents the years before pandemic-related measures, such as lockdowns in the state, which could significantly impact the results.

The data were imported from the DATASUS website and organized using Excel Office 2016 software. The mortality rate was calculated by dividing the number of deaths (numerator) by the resident population in the state (denominator) for each year (2009 to 2020), multiplying each result by 100,000 inhabitants.

Data analysis was conducted in several stages. First, mortality rates were transformed into base-10 logarithms for better regression adjustment. Next, the Prais–Winsten linear regression model was applied to determine the temporal trend of mortality rates during the study period. This included estimating the angular coefficient (β), *p*-value, and predictive capacity (r^2^) of the model, stratified by age group and sex. Variables in which the “ignored” category had a higher prevalence than any of the valid categories were excluded from the analysis to ensure the robustness of the results. Finally, the annual percentage change (APC) and their respective confidence intervals (95% CI) were calculated. Mortality rate trends were interpreted as increasing (*p* < 0.05 and positive β), decreasing (*p* < 0.05 and negative β), or stable (*p* ≥0.05) [13]. The variables were analyzed using the Stata 13.0 software.

Since this study used secondary data, which were freely and publicly available, there was no need for review by a Research Ethics Committee involving human subjects, according to Resolution 510/2016 of the Brazilian Ministry of Health.

## 3. Results

The temporal trend of pedestrian mortality for males and females aged from 0 to over 80 years was analyzed, stratified by age group during the period from 2009 to 2020 in the state of Espírito Santo, Brazil. A decreasing trend in the overall behavior was observed, but significant variations were identified when analyzed separately.

Table 1 presents the sociodemographic variables analyzed, comprising a total of 1969 pedestrians who died due to traffic accidents. There was a predominance of males (75%), mixed race (57.4%), individuals with 1 to 7 years of formal education (66%), and unmarried individuals (72%).

The annual percentage variation in pedestrian mortality by age group showed a decreasing trend in almost all age groups, except for the 80 years and older group. A sharper decline was noted in the 0 to 4 years age group, with a reduction of 20.57%, followed by the 5 to 9 years and 25 to 29 years groups, both showing reductions of 14.89% (Table 2).

Table 3 presents the annual percentage variation in mortality for male pedestrians. A decreasing trend was observed across most age groups, ranging from 2.28% to 16.82%. However, the age groups of 0–4 years and 80 years or more remained stationary throughout the period. The steepest decline occurred in the 25–29 years group, followed by the 20–24 years group.

Regarding women, Table 4 reveals predominantly decreasing trends across most age groups, indicating stable or declining mortality rates between 2009 and 2020. Statistically significant downward trends were identified in the following four specific age groups: 20–24 years (APC = −16.82%), 50–54 years (APC = −12.9%), 55–59 years (APC = −10.87%), and 60–64 years (APC = −14.89%). Additionally, the 65–69 age group also exhibited a decreasing trend (APC = −10.87%;). Overall, the total female mortality trend showed a statistically significant decline over the study period (APC = −8.8%).

**Figure 2:**
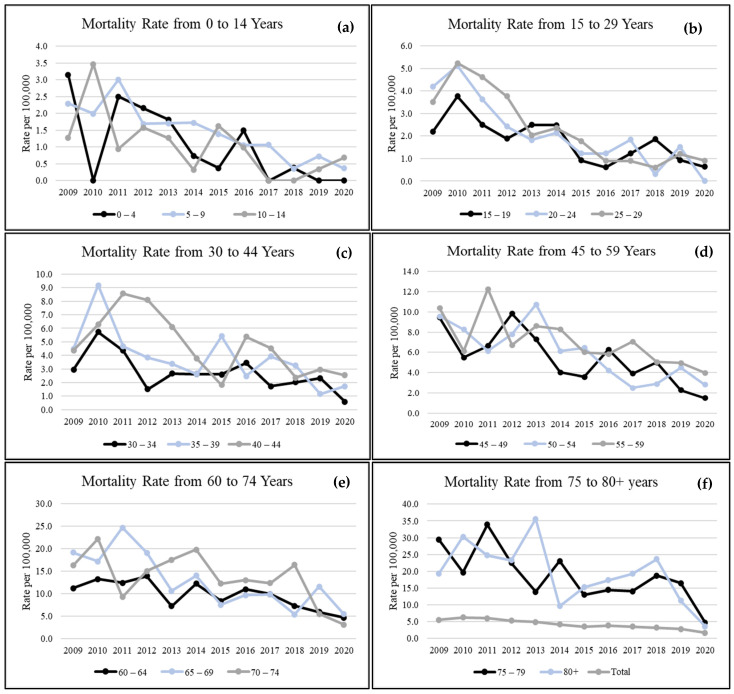
General pedestrian mortality rate stratified by age: (**a**) mortality rate from 0 to 14 years; (**b**) mortality rate from 15 to 29 years; (**c**) mortality rate from 30 to 44 years; (**d**) mortality rate from 45 to 59 years; (**e**) mortality rate from 60 to 74 years; (**f**) mortality rate for age 75 years and older.

Figure 2 displays descriptive data on mortality rates stratified by age group in graphs spanning the period from 2009 to 2020. A decline in mortality rates over the years was observed across all age groups studied. For individuals aged 0 to 14 years, rates remained consistently low, ranging from 0.1 to 3.5 per 100,000 inhabitants. In contrast, age groups 60 years and older exhibited higher initial rates, exceeding 10 per 100,000 inhabitants, with a notable peak in 2013 for those aged 80 years or more. These findings highlight that older age groups, especially those above 60 years, maintain higher mortality rates throughout the period, emphasizing their vulnerability compared to younger individuals.

**Figure 3:**
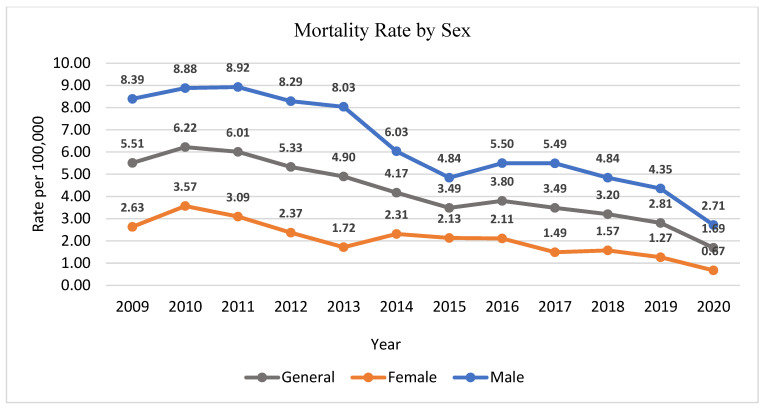
General pedestrian mortality rate and by sex.

In Figure 3, the pedestrian mortality data in Brazil, stratified by sex between 2009 and 2020, show an overall declining trend in the mortality rate for both men and women. It is observed that, in 2009, the overall mortality rate was 5.51 per 100,000 inhabitants, decreasing to 1.69 in 2020. During the same period, male mortality decreased from 8.39 to 2.71, while female mortality dropped from 2.63 to 0.67.

## 4. Discussion

The present study evaluated the pedestrian mortality rate between 2009 and 2020 in the state of Espírito Santo, observing an overall decrease in mortality during this period for both men and women. These findings are supported by a study conducted by Santiago and colleagues (2023) during the period from 2010 to 2019 in Latin America and the Caribbean, which showed a 6.26% reduction in traffic accident mortality rates. However, this region remained at the highest mortality rate for traffic accidents in the 2019 “Global Burden of Disease Study” (GBD) [14]. Thus, while there has been a global reduction in traffic accident mortality rates, the local reality of these incidents remains a severe public health issue.

Regarding traffic accidents (TAs), a study conducted in 2024 in Colombia (South America) from 2008 to 2021 showed a rising trend in TAs in urban areas (74.1%) compared to rural areas (25.9%) [15]. This suggests that the growing vehicle fleet and urbanization must be met with effective legislation to address the prevalent TAs in urban cities.

Despite this study showing positive progress in reducing mortality rates, the situation in regions and countries with low socioeconomic development is concerning. According to the World Health Organization (WHO), 90% of traffic accident deaths occur in developing countries [16]. This highlights the importance of a country’s economy and its influence on the occurrence of traffic accidents [17]. A global study analyzing 181 countries showed that GDP positively impacted pedestrian death reductions, suggesting an association between mortality rates and a country’s income. Higher GDP is typically associated with better urban development and infrastructure investments, ensuring safer streets for pedestrians through wider sidewalks, overpasses, proper signage, and efficient public transportation systems [17].

A study by Leitão et al. (2022) analyzed factors associated with traffic accidents, revealing that pedestrian mortality rates were highest in Montenegro, European Union [18]. However, following the implementation of safety campaigns for road users, there was a downward trend. Similarly, Brazil shows a downward trend in traffic accident mortality rates, as it has been developing policies to address this issue [18]. However, this relationship is not necessarily linear and can be influenced by a variety of other factors, including road safety policies, traffic culture, demographic profile, and law enforcement. As a developing country, Brazil faces unique challenges in this context, where improving infrastructure and implementing effective road safety policies have contributed to reducing pedestrian mortality.

In this study, there was a significant decrease in mortality rates across almost all age groups in Espírito Santo. The data show that the pedestrian mortality rate for all ages, except for those aged 80 and over, showed a significant downward trend, as indicated by the Annual Percent Change (APC) values. The greatest declines in mortality rates were observed among children aged 0–4 years (−20.57%) and young adults aged 20–24 years (−16.82%). This decline can be attributed to various preventive and traffic safety policies implemented over the years.

However, it is important to note that the reduction in mortality was not uniform across all age groups. The pedestrian mortality rate for those aged 80 years or more showed a stationary trend, indicating that prevention policies may not have been as effective for this specific group. Mortality rates are higher in older age groups compared to younger ones, as illustrated in Figure 1. This trend is evident in both males and females.

These data suggest vulnerability among older pedestrians and the need for additional focused strategies, as aging is considered a vulnerability factor for most elderly individuals over 60 years old who have greater difficulties crossing streets [19]. Age-related cognitive decline, as well as slower gait and reduced visual acuity, are risk factors for collisions with vehicles, as faster walking speed and proper vehicle visibility are necessary for safe crossing. Therefore, age-related factors indicate that challenges must be addressed to ensure the safety of all pedestrians [20,21].

Another factor observed in this study is the predominance of male deaths, with 1483 individuals (75%) among the fatalities. Furthermore, when comparing mortality rates between sexes, it is evident that males exhibit higher mortality rates across all age groups and the study period, suggesting greater vulnerability among men in pedestrian-related accidents. The World Health Organization (WHO) identifies men as a higher-risk group for traffic fatalities, with rates three times higher than those of women [16]. This discrepancy may be linked to male behavior, as men are more likely to violate traffic rules than women [22]. A study by Kayvan Aghabayk and colleagues (2021) highlighted that men behave differently from women when crossing streets, initiating crossings before the traffic signal permits. Another factor to consider is the use of technology by pedestrians, which, regardless of type, leads to risky behaviors due to distraction, increasing the likelihood of accidents [23].

When examining mortality rates by age group and sex, there is significant variation in mortality across different age groups. Pedestrian mortality data in Espírito Santo from 2009 to 2020 show a clear decreasing trend for both men and women. In 2009, the general mortality rate was 5.51 per 100,000 inhabitants, dropping to 1.69 in 2020. Studies show similar trends for Brazil and the southeastern region of the country.

A study by Fernandes et al. (2019) analyzed pedestrian mortality in Brazil from 1996 to 2015 and found a 70.7% decrease in male pedestrian fatalities and a 26.37% decrease in female fatalities in the southeastern region [24]. In Espírito Santo, there was an 8.8% reduction overall. In terms of age groups, males showed the greatest reduction between ages 0–4 and 20–29 years, while females had the largest reduction between ages 20–24 and 60–64 years.

Regarding the impact of public policies, the 17 Sustainable Development Goals (SDGs) established by the United Nations are considered an important tool to address the issue, aiming to develop and implement public policies for human well-being. In this context, reducing traffic-related fatalities is part of the SDG goal set for 2011–2030. However, a study by Moreira et al. (2017) found that, in the last 20 years, Brazil’s traffic fatalities have remained stable, with an average of 39,000 deaths per year from TAs, 13,200 of which were adolescents and young adults aged 10–29 [25]. This study indicates an instability with both upward and downward trends over time, showing that, despite public policy development, Brazil struggles to meet the SDG target [25].

Although this study presents the lowest mortality rates in 2020, it should be considered that the reduction in mortality for that specific year may be influenced by the epidemiological scenario caused by the COVID-19 pandemic, which led to preventive health measures, such as yellow alert status, moderate-risk signals, and lockdowns. This reduced movement of people had a direct impact on the pedestrian mortality rate from traffic accidents.

This study presents certain limitations that should be considered when interpreting the findings. This analysis relied on secondary data from the Mortality Information System, which may lead to an underestimation of the actual number of pedestrian traffic fatalities. Furthermore, the results are specific to Espírito Santo and may not be generalizable to regions with different socioeconomic and infrastructural contexts. These limitations underscore the importance of cautious interpretation and the need for further research to validate and expand upon these findings.

Nevertheless, the findings of this study highlight the critical role of public policies and legislation in preventing pedestrian mortality in traffic. To ensure that these measures are both effective and contextually appropriate, it is essential to deepen the understanding of the risk factors associated with these events. Therefore, future studies should explore sociodemographic, behavioral, and environmental determinants of pedestrian deaths in order to inform more targeted and evidence-based interventions.

In this context, it is necessary to adopt a broad, intersectoral approach that goes beyond formal legislation by incorporating urban infrastructure improvements, ongoing educational campaigns, and more effective enforcement mechanisms. The integration of these components is crucial to fostering safer and more accessible urban environments and to sustainably reducing risks to pedestrian life, particularly among the most vulnerable populations.

## 5. Conclusions

This study presented a reduction in the mortality rate over the period of 2009–2020 in the state of Espírito Santo, especially among children and young adults, suggesting progress in public policies aimed at traffic safety. However, mortality rates remained stable among the elderly, suggesting the need for more targeted policies for this vulnerable group.

## Figures and Tables

**Table 1:** Sociodemographic characteristics of the pedestrians studied, Brazil, 2020.

Variables	n	(%)
Gender		
Female	486	25%
Male	1483	75%
Race/Color ^#^		
White	632	35%
Black	135	7.5%
Asian	1	0.1%
Mixed Race	1033	57.4%
Indigenous	0	0%
Education ^#^		
None	108	13%
1 to 3 years	271	33%
4 to 7 years	269	33%
8 to 11 years	146	18%
12+ years	33	4%
Martial Status ^#^		
Partnered	534	28%
Single	1361	72%

**^#^** Unreported data were excluded from each category.

**Table 2:** Temporal trend analysis of pedestrian mortality by age group from 2009 to 2020.

Variables	APC%(95%CI)	Mortality Rate per 100,000	*p*-Value	Trend
0 to 4 years	−20.57 (−32.39; −6.67)	1.07	0.013 *	Decreasing
5 to 9 years	−14.89 (−18.72; −10.87)	1.47	*p* < 0.001 *	Decreasing
10 to 14 years	−10.87 (−18.72; −2.28)	1.05	0.010 *	Decreasing
15 to 19 years	−10.87 (−18.72; −2.28)	1.79	0.009 *	Decreasing
20 to 24 years	−16.82 (−22.38; −12.9)	2.12	*p* < 0.001 *	Decreasing
25 to 29 years	−14.89 (−20.57; −8.8)	2.34	*p* < 0.001 *	Decreasing
30 to 34 years	−8.8 (−16.82; −1.83)	2.68	0.020 *	Decreasing
35 to 39 years	−8.8 (−14.89; −4.5)	3.72	0.001 *	Decreasing
40 to 44 years	−6.67 (−14.89; −0.46)	4.63	0.037 *	Decreasing
45 to 49 years	−10.87 (−16.82; −4.5)	5.35	0.004 *	Decreasing
50 to 54 years	−8.8 (−14.89; −4.5)	5.85	0.002 *	Decreasing
55 to 59 years	−4.5 (−6.67; −2.28)	6.90	*p* < 0.001 *	Decreasing
60 to 64 years	−6.67 (−8.8; −2.28)	9.37	0.002 *	Decreasing
65 to 69 years	−8.8 (−12.9; −6.67)	11.90	*p* < 0.001 *	Decreasing
70 to 74 years	−8.8 (−18.72; −0.16)	12.99	0.047 *	Decreasing
75 to 79 years	−8.8 (−14.89; −2.28)	18.04	0.007 *	Decreasing
80+ years	−8.8 (−18.72; 0.21)	18.51	0.054	Stationary
Total	−8.8 (−12.9; −4.5)	4.17	*p* < 0.001 *	Decreasing

* *p* < 0,05; APC = annual percent change; 95% CI = 95% confidence interval.

**Table 3:** Temporal trend analysis of male pedestrian mortality by age group from 2009 to 2020.

Variables	APC%(95%CI)	Mortality Rate per 100,000	*p*-Value	Trend
0 to 4 years	−14.89 (−38.34; 12.2)	1.20	0.200	Stationary
5 to 9 years	−10.87 (−14.89; −4.5)	1.81	0.001 *	Decreasing
10 to 14 years	−10.87 (−20.57; −2.28)	1.44	0.023 *	Decreasing
15 to 19 years	−10.87 (−18.72; −2.28)	2.65	0.007 *	Decreasing
20 to 24 years	−14.89 (−20.57; −8.8)	3.37	*p* < 0.001 *	Decreasing
25 to 29 years	−16.82 (−22.38; −10.87)	3.72	*p* < 0.001 *	Decreasing
30 to 34 years	−4.5 (−8.8; −0.23)	4.11	0.041 *	Decreasing
35 to 39 years	−8.8 (−14.89; −4.5)	6.20	*p* < 0.001 *	Decreasing
40 to 44 years	−10.87 (−20.57; −1.37)	7.56	0.031 *	Decreasing
45 to 49 years	−10.87 (−16.82; −4.5)	8.75	0.001 *	Decreasing
50 to 54 years	−8.8 (−16.82; −1.83)	9.17	0.020 *	Decreasing
55 to 59 years	−4.5 (−6.67; −0.46)	10.57	0.029 *	Decreasing
60 to 64 years	−2.28 (−6.67; −0.23)	14.36	0.033 *	Decreasing
65 to 69 years	−8.8 (−14.89; −4.5)	17.66	0.002 *	Decreasing
70 to 74 years	−10.87 (−20.57; −0.92)	19.94	0.036 *	Decreasing
75 to 79 years	−8.8 (−12.9; −2.28)	29.46	0.001 *	Decreasing
80+ years	−4.5 (−12.9; 2.33)	33.24	0.153	Stationary
Total	−8.8 (−10.87; −4.5)	6.29	*p* < 0.001 *	Decreasing

* *p* < 0,05; APC = annual percent change; 95% CI = 95% confidence interval.

**Table 4:** Temporal trend analysis of female pedestrian mortality by age group from 2009 to 2020.

Variables	APC%(95%CI)	Mortality Rate per 100,000	*p*-Value	Trend
0 to 4 years	−10.87 (−22.02; 0.23)	0.94	0.054 *	Stationary
5 to 9 years	-	1.1	-	-
10 to 14 years	−8.8 (−32.39; 17.49)	0.66	0.386	Stationary
15 to 19 years	−6.67 (−16.82; 4.71)	0.90	0.209	Stationary
20 to 24 years	−16.82 (−25.87; −25.87)	0.83	0.005 *	Decreasing
25 to 29 years	−8.8 (−22.38; 2.33)	0.90	0.114	Stationary
30 to 34 years	−4.5 (−18.72; 7.15)	1.21	0.354	Stationary
35 to 39 years	−2.28 (−18.72; 12.2)	1.20	0.596	Stationary
40 to 44 years	4.71 (−0.46; 9.65)	1.70	0.069	Stationary
45 to 49 years	−2.05 (−6.67; 2.33)	1.98	0.389	Stationary
50 to 54 years	−12.9 (−24.14; −1.37)	2.60	0.032 *	Decreasing
55 to 59 years	−10.87 (−16.82; −4.5)	3.37	0.004 *	Decreasing
60 to 64 years	−14.89 (−22.38; −6.67)	4.71	0.001 *	Decreasing
65 to 69 years	−10.87 (−14.89; −4.5)	6.86	*p* < 0.001 *	Decreasing
70 to 74 years	−6.67 (−16.82; 4.71)	7.21	0.203	Stationary
75 to 79 years	−1.6 (−12.9; 9.65)	9.57	0.153	Stationary
80+ years	−10.87 (−25.87; 4.71)	8.96	0.146	Stationary
Total	−8.8 (−14.89; −4.5)	2.05	0.001 *	Decreasing

* *p* < 0,05; APC = annual percent change; 95% CI = 95% confidence interval.

## Data Availability

The data from the article are publicly available and can be retrieved from the DATASUS platform https://datasus.saude.gov.br/informacoes-de-saude-tabnet/ (accessed on 5 March 2024). Additionally, the authors are willing to provide the .dta file upon request if necessary.
